# Machine Learning-Based Analysis of Large-Scale Transcriptomic Data Identifies Core Genes Associated with Multi-Drug Resistance

**DOI:** 10.3390/ijms27031245

**Published:** 2026-01-27

**Authors:** Yanwen Wang, Fa Si, Lei Huang, Zhengtai Li, Changyuan Yu

**Affiliations:** College of Life Science and Technology, Beijing University of Chemical Technology, Beijing 100029, China; yanwenwang23@126.com (Y.W.); sifa2001@163.com (F.S.); 2022410035@buct.edu.cn (L.H.); lizhengtaii@126.com (Z.L.)

**Keywords:** cellular omics, feature importance, gene and pathway function, drug resistance mechanisms

## Abstract

Drug resistance is an important challenge in medical research and clinical practice, posing a serious threat to the effectiveness of current therapeutic strategies. Transcriptomics has played a crucial role in analyzing resistance-related genes and pathways, while the application of machine learning in high-throughput data analysis and prediction has also opened up new avenues in this field. However, existing studies mostly focus on a single drug or specific categories, and their conclusions are limited in applicability across drug categories, while studies on drugs beyond antibacterial and antitumor categories remain limited. In this study, we systematically analyzed the transcriptomic data of resistant cell lines treated with 1738 drugs spanning 82 categories and identified core genes through an integrated analysis of three classical machine learning methods. Using the antibacterial drug salinomycin as an example, we established a resistance prediction model that demonstrated high predictive accuracy, indicating the significant value of the selected core genes in prediction. Meanwhile, some of the core genes identified through the protein–protein interaction (PPI) network overlapped with those derived from machine learning analysis, further supporting the reliability of these core genes. Pathway enrichment analysis of differential genes revealed potential resistance mechanisms. This study provides a new perspective for exploring resistance mechanisms across drug categories and highlights potential directions for resistance intervention strategies and novel drug development.

## 1. Introduction

Drug resistance refers to the phenomenon in which pathogens, tumor cells, or other target cells become less sensitive or unresponsive to therapeutic agents after long-term or repeated exposure, leading to reduced treatment efficacy or therapeutic failure. Drug resistance is widely recognized as one of the major challenges in clinical treatment and drug development [1,2]. During cancer chemotherapy, resistance often leads to a significant decline in efficacy [3], while in the treatment of infectious diseases, resistance contributes to the global challenge of antibiotic resistance and has become a critical global public health challenge [4]. Despite extensive research on drug resistance mechanisms, the problem remains prevalent and increasingly serious. In recent years, epidemiological data have further shown that the treatment failure rate and mortality risk related to resistance continue to increase [5,6], which imposes a substantial burden on patients’ quality of life and healthcare systems. Due to its complex causes and considerable heterogeneity, the study of resistance mechanisms is not only an important part of understanding disease progression but also a key approach to promoting new drug development and optimizing treatment strategies [2,7].

At present, academic research primarily elucidates the molecular mechanisms underlying drug resistance from several perspectives, including drug efflux mechanisms [8,9], DNA damage repair and apoptosis evasion [10,11], epigenetic regulation [12], as well as microenvironmental adaptation and metabolic regulation [13,14]. These studies have provided important theoretical foundations for understanding drug resistance and have laid the groundwork for resistance-reversal strategies that are now progressing into experimental or even clinical stages [15]. Systematic comparative analyses across drug classes are essential for identifying shared molecular determinants of resistance. Chifiriuc et al. explored the common mechanisms between antimicrobial and anticancer agents, highlighting their similarities in drug targets, modes of action, and resistance pathways [16]. However, most existing studies remain focused on single drugs or limited categories, and their findings have limited generalizability across classes. Existing literature has predominantly focused on antimicrobial and anticancer drugs [17,18], whereas resistance mechanisms in other drug categories and systematic cross-class comparisons remain underexplored.

With the development of high-throughput sequencing technology, transcriptomics provides a systematic approach to analyzing the dynamic molecular responses of cells to drug treatment [19,20]. Multiple studies have shown that through comprehensive analysis of gene expression profiles, researchers can identify resistance-related genes and pathways, thereby providing a global view of the underlying molecular mechanisms [21,22]. In recent years, machine learning methods, owing to their strengths in high-dimensional data processing and pattern recognition, have been gradually applied to drug response prediction and resistance research [23,24]. In the field of antibacterial drugs, they have been widely applied to predict bacterial resistance phenotypes [25], while in the field of cancer, models combined with gene expression profiles have also demonstrated good performance in predicting drug sensitivity [26]. Integrating statistical methods, machine learning algorithms, and functional enrichment analysis is expected to provide a multi-dimensional framework for analyzing resistance phenomena from both data- and mechanism-oriented perspectives [27]. Accordingly, this study systematically collected and analyzed transcriptomic data of cells under treatment with thousands of drugs, with broad coverage and sufficient data volume. Within a cross-drug-category framework, we conducted a systematic comparison of resistance mechanisms and explored their potential shared molecular patterns. In the analysis process, we implemented a sequential research framework that integrates statistical and biological evidence, progressing from differential gene identification and core gene screening to classification modeling, network construction, and and Gene Ontology (GO) and Kyoto Encyclopedia of Genes and Genomes (KEGG) enrichment analysis. Through this strategy, we aim to advance understanding of the systematic characteristics of resistance mechanisms. This approach also provides theoretical support for the development of anti-resistance targets and individualized therapeutic strategies.

## 2. Results

### 2.1. Drug Categories and Analytical Workflow

**Figure 1:**
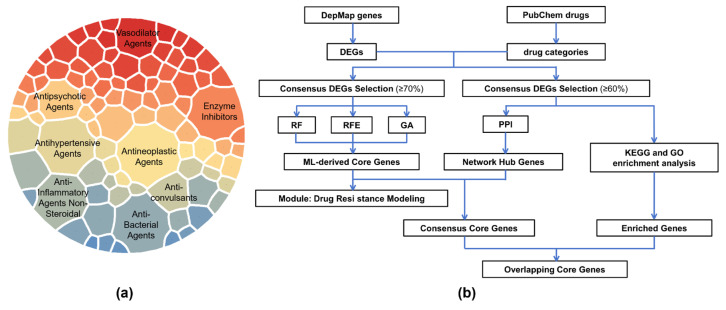
Drug classification and study workflow. (**a**) Distribution of drug categories, showing the eight categories with the largest numbers of compounds. The area of each sector is proportional to the number of drugs. (**b**) Overview of the study workflow, starting with drug categorization and differential gene expression identification, followed by core gene selection, resistance modeling, and functional enrichment analyses.

The distribution of drug categories and the overall analytical workflow of this study are presented in Figure 1. The lineage composition of resistant cells for each drug is summarized in Supplementary Table S1, providing an overview of tissue representation across the resistant populations, which are relatively balanced across lineages. The lineage composition of resistant cells for each drug is summarized in Supplementary Table S1, and that of sensitive cells is provided in Supplementary Table S2. Overall, tissue representation across lineages is generally balanced, with only a few extreme cases dominated by one or two lineages (see also Supplementary Figures S1 and S2).

### 2.2. Differential Gene Expression Landscape

**Figure 2:**
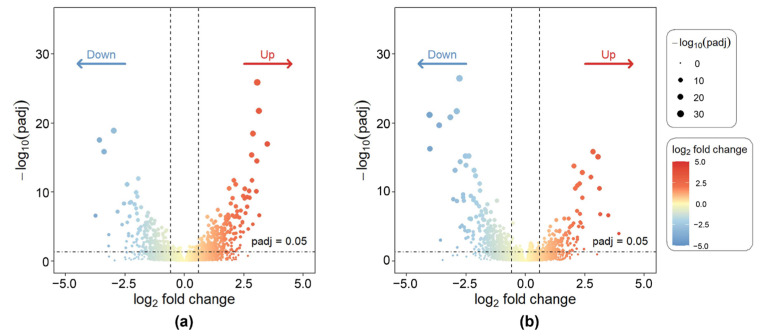
Volcano plots of DEGs for representative drug-treated cell lines. (**a**) Miltefosine; (**b**) Tylosin.

Through differential expression analysis of 1738 drug-treated cell line samples, gene expression alterations associated with drug resistance were effectively identified, thereby narrowing the pool of candidate genes and providing a foundation for subsequent core gene selection. Representative results are shown in Figure 2, which displays volcano plots of differentially expressed genes for the antitumor drug miltefosine and the antibacterial drug tylosin, respectively. The volcano plots revealed a clear distribution of differentially expressed genes (DEGs), with significant upregulated and downregulated genes distinctly separated, and a well-defined stratification between statistical significance and fold-change magnitude.

**Figure 3:**
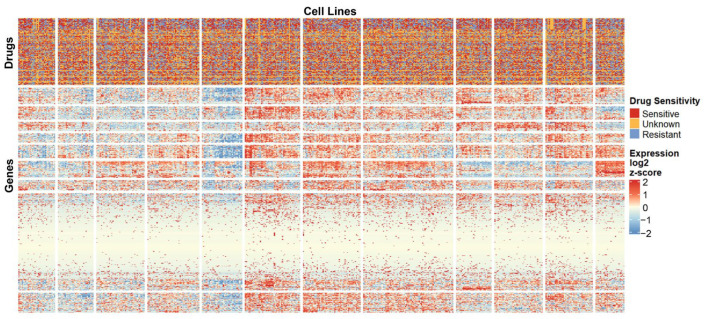
Heatmap of gene expression for the Antibacterial Agents category. A subset of genes and cell lines is shown to illustrate overall expression patterns and clustering. Top annotations indicate drug sensitivity for each cell line.

Figure 3 presents a heatmap of gene expression for the Antibacterial Agents category. Sample annotations at the top correspond to drug sensitivity information for all agents within this category. The clustering patterns in the heatmap were well defined. The heatmap results demonstrate distinct clustering patterns of gene expression across different samples. Although the high- and low-expression regions are not completely separated, likely due to the data complexity, several gene modules with similar expression profiles and sample groups sharing comparable molecular features can still be identified. Notably, the gene cluster located in the upper-right region exhibits pronounced upregulation, while multiple modules in the central-left area show marked downregulation. By contrast, genes within the lower-central modules display minimal inter-sample variation, suggesting a weaker transcriptional response to drug treatment in these regions.

### 2.3. Analysis of Machine Learning-Based Core Gene Selection and Resistance Classification Modeling

#### 2.3.1. Threshold-Based Identification of Shared DEGs

**Figure 4:**
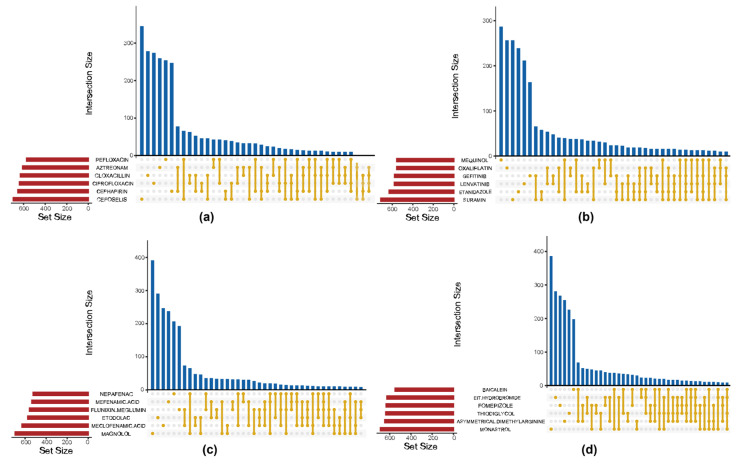
Venn diagrams of intersecting DEGs across representative drug categories. (**a**) Antibacterial Agents; (**b**) Antineoplastic Agents; (**c**) Anti-Inflammatory Agents; (**d**) Non-Steroidal Enzyme Inhibitors.

Figure 4 presents Venn diagrams illustrating the top six drugs with the highest intersection frequencies within the Antibacterial Agents, Antineoplastic Agents, Anti-Inflammatory Agents, and Non-Steroidal Enzyme Inhibitors categories, respectively. A strict complete intersection across drugs yielded only a minimal set of shared genes, indicating that cellular resistance responses to different compounds within the same drug class may involve distinct and drug-specific regulatory mechanisms. Nevertheless, a certain degree of overlap was still observed among drugs within each category, suggesting the existence of common response modules induced by similar types of compounds. To better capture these shared signatures, a thresholded intersection criterion of 70% was applied, whereby genes appearing in at least 70% of differential gene lists within a given drug category were retained. This threshold ensures a sufficiently large and representative set of genes for feature selection while avoiding overly stringent filtering that would exclude relevant genes. This approach expanded the pool of candidate genes while preserving consistency across compounds, effectively reducing noise from drug-specific variability.

#### 2.3.2. Machine Learning-Derived Core Drug Resistance Genes

**Figure 5:**
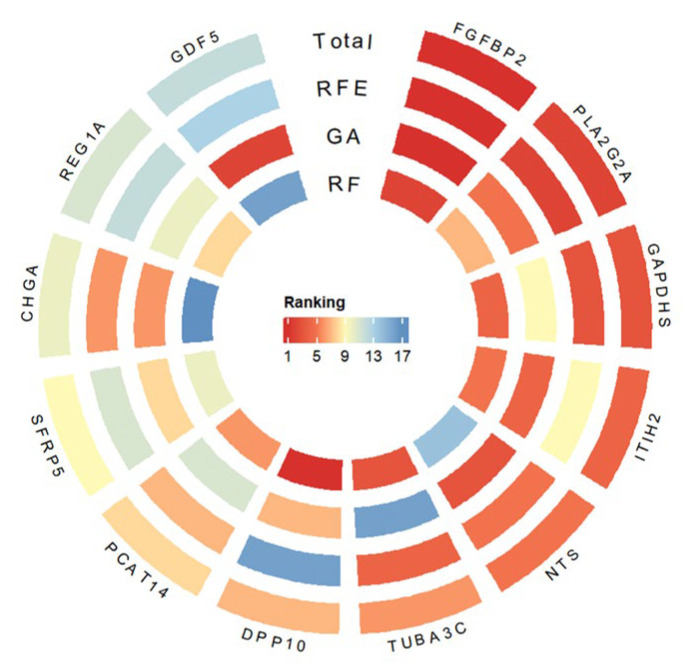
Representative subset of genes visualized according to their importance scores derived from integrated feature selection.

Three classical feature selection methods—Random Forest, Genetic Algorithm, and Recursive Feature Elimination (RFE)—were applied to prioritize intersected DEGs and identify core resistance-related genes. The results from all three approaches were integrated using a weighted average, yielding a robust ranking of gene importance. Figure 5 presents MILTEFOSINE as a representative drug to demonstrate the gene-ranking results. In response to MILTEFOSINE treatment, FGFBP2 and PLA2G2A consistently ranked among the top candidates across all feature selection methods and GAPDHS rankings, suggesting their involvement in the molecular mechanisms underlying drug resistance.

### 2.4. Performance of Resistance Classification Models

Through machine learning-driven feature selection, irrelevant and redundant genes were eliminated, reducing data dimensionality. The selected core genes were incorporated into a random forest classifier to construct the resistance prediction model.

**Figure 6:**
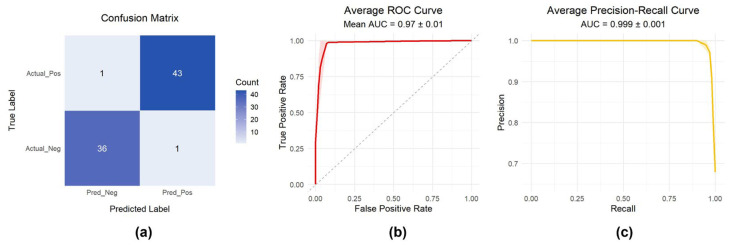
Model performance evaluation. (**a**) Confusion matrix of classification results; (**b**) Receiver Operating Characteristic (ROC) curve; (**c**) Precision–Recall (PR) curve.

The model achieved excellent classification performance (Figure 6a–c), with the confusion matrix (Figure 6a) confirming high classification accuracy. The ROC curve (Figure 6b) yielded an AUC of 0.97 ± 0.01, and the PR curve (Figure 6c) achieved an AUC of 0.999 ± 0.001. To provide a broader view of resistance prediction across drugs from different pharmacological categories, the corresponding results for Oxaliplatin (Antineoplastic Agents), Nafcillin (Antibacterial Agents), Methotrexate (Enzyme Inhibitors), and Phenylbutazone (Non-Steroidal Anti-Inflammatory Agents) are presented in Supplementary Figure S3.

### 2.5. PPI Network Construction

**Figure 7:**
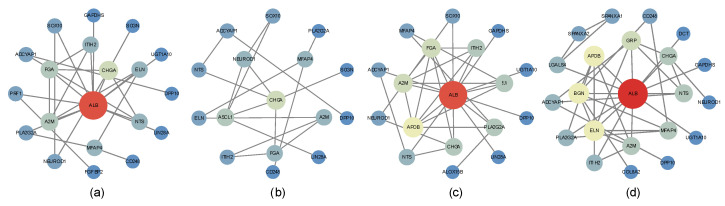
PPI networks of representative drug categories. Node size and color correspond to the degree of connectivity. Only genes appearing in at least 60% of drugs within each category were included. This cutoff was chosen as a balance, since stricter thresholds would retain too few genes, while more relaxed thresholds would include weak or inconsistent genes, potentially increasing noise and reducing interpretability. The plots display PPI networks of (**a**) antibacterial agents, (**b**) antineoplastic agents, (**c**) non-steroidal anti-inflammatory agents (NSAIDs), and (**d**) enzyme inhibitors.

Figure 7 displays the PPI networks of antibacterial agents, antineoplastic agents, non-steroidal agents, and enzyme inhibitors, where node size and color reflect the degree values. Core genes were identified based on betweenness centrality (Table 1), and the complete node-level metrics for all PPI networks are provided in Supplementary Table S3. Notably, several genes were consistently detected by both network analysis and the machine learning feature importance method, including NTS and PLA2G2A (antibacterial agents), A2M and NTS (antineoplastic agents), ELN, ITIH2, ADCYAP1, NTS, and GAPDHS (non-steroidal agents), and ADCYAP1, NTS, CHGA, and PLA2G2A (enzyme inhibitors).

### 2.6. GO Terms and KEGG Pathways Enriched by DEGs

**Figure 8:**
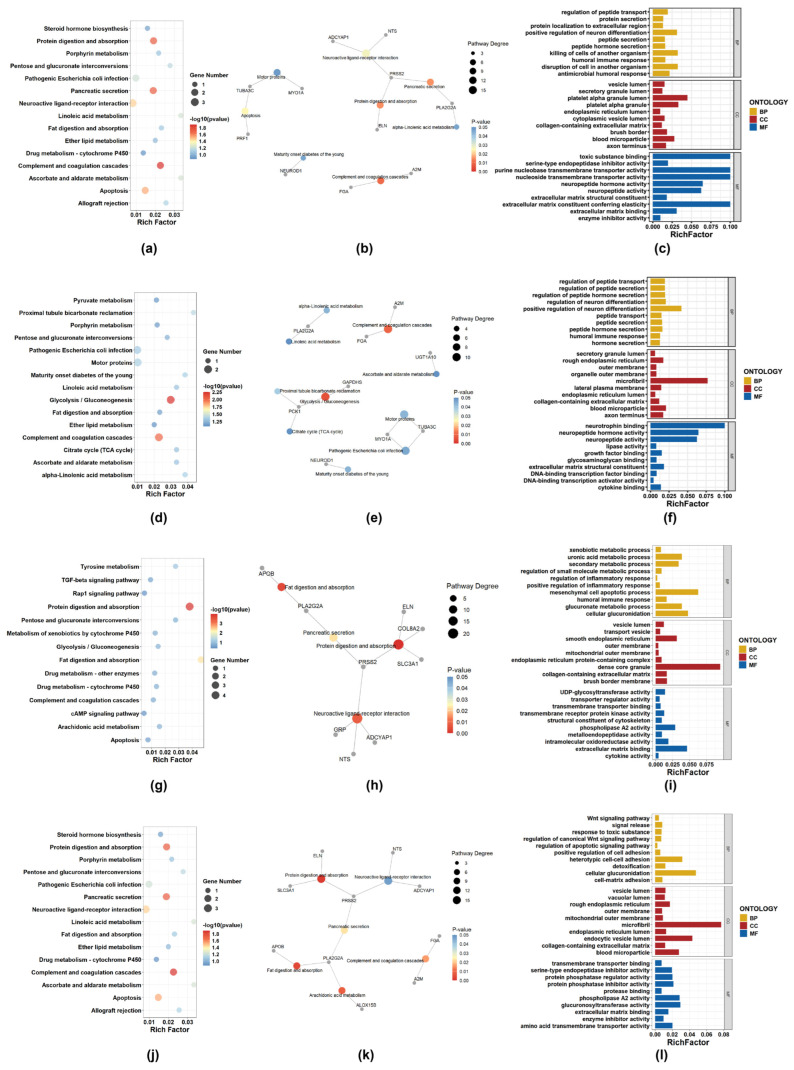
For each category, the top 15 KEGG pathways (bubble plot and network diagram) and the top 10 GO terms from each domain (BP, CC, and MF; bar charts) are shown. (**a**–**c**) Antibacterial Agents; (**d**–**f**) Antineoplastic Agents; (**g**–**i**) Non-steroidal anti-inflammatory agents (NSAIDs); (**j**–**l**) Enzyme inhibitors.

Figure 8 presents the KEGG and GO analysis results for four drug categories: Antibacterial Agents, Antineoplastic Agents, Anti-Inflammatory Agents, Non-Steroidal, and Enzyme Inhibitors. Due to the relatively small number of intersecting genes, to obtain a sufficient number of pathways for visualization, a strict *p*-value cutoff was not applied, but only pathways with relatively high significance and enrichment ratios were prioritized.

In the KEGG enrichment analysis, the complement and coagulation cascades pathway was consistently enriched across Antibacterial Agents, Antineoplastic Agents, and Enzyme Inhibitors, involving key genes FGA and A2M, both of which are closely related to coagulation and inflammatory processes. This cross-category enrichment suggests that alterations in coagulation-related pathways, extracellular matrix remodeling, or inflammation-associated responses may represent shared adaptive features underlying drug resistance across distinct pharmacological classes.

Antineoplastic Agents were additionally characterized by significant enrichment in the Glycolysis/Gluconeogenesis pathway, with differential expression of metabolic genes including PCK1 and GAPDHS, indicating potential metabolic reprogramming associated with resistance development and adaptation to long-term therapeutic stress. In Non-Steroidal Anti-Inflammatory Agent-resistant cell lines, enrichment of the Neuroactive ligand–receptor interaction pathway was observed, involving signaling-related genes such as NTS, ADCYAP1, and GRP, suggesting that dysregulation of receptor-mediated signaling and cellular communication may contribute to drug tolerance. By contrast, Enzyme Inhibitors were mainly associated with pathways related to Arachidonic acid metabolism and Protein digestion and absorption, reflecting distinct metabolic and signaling adaptations in response to enzyme-targeted therapies.

The KEGG pathway network diagrams show pathways with *p*-value < 0.05 and the intersecting differentially expressed genes enriched in these pathways. The GO analysis figures display the top 10 enriched terms in BP, CC, and MF. These results are intended to provide an exploratory overview of the potential biological functions underlying the observed gene expression changes.

## 3. Discussion

This study systematically analyzed the transcriptomic profiles of cell lines treated with diverse pharmacological agents and identified shared DEGs across pharmacological categories. Volcano plots and heatmaps revealed that drug exposure induced convergent transcriptional effects, suggesting potential links between drug mechanisms of action and associated gene expression alterations, and implying shared molecular response pathways among different treatments. Building on these intersecting DEGs, multiple machine learning algorithms were integrated for feature selection, identifying core genes closely associated with drug resistance and constructing a resistance classification model based on these core genes. This framework reduces model- and drug-specific biases, enabling robust identification of key resistance genes with potential generalizability to untested compounds.

Compared with traditional modeling approaches that rely on the full gene set, strategies based on core genes demonstrate clear advantages. Dimensionality reduction not only enhances model stability and robustness under small-sample conditions but also reduces the risk of overfitting. Since the selection of core genes spanned multiple drug categories, the resulting gene set captured common features within compound classes. This enhanced its adaptability and generalizability when applied to untested drugs of the same type. This strategy further validates the critical role of core genes in resistance prediction and provides a solid basis for in-depth investigation of resistance mechanisms and potential targeted intervention strategies. In addition, this approach improves computational efficiency and interpretability while offering biological insights into the underlying mechanisms of resistance.

After the core genes were screened, a PPI network was constructed and integrated with KEGG and GO enrichment analyses to verify the functional connections and enhance the biological interpretability of the integrated results. The results showed that core genes across different drug categories are likely involved in signal transduction, inflammatory regulation, and extracellular matrix remodeling. For example, PLA2G2A may participate in inflammation control and tissue regeneration; A2M is linked to the inhibition of the PI3K/AKT signaling pathway; and ITIH2 may contribute to extracellular matrix stabilization and tumor progression. These core genes may serve as key molecular determinants in the development of drug resistance and contribute to a common explanatory framework for resistance mechanisms among various drug types. Among them, PLA2G2A served as an important connecting node in the enriched pathway networks of all four drug categories, highlighting its potential role as a mechanistic hub mediating multidrug resistance through modulation of inflammation and tissue remodeling. Our results reveal that their coordinated regulation occurs consistently across multiple pharmacological classes, highlighting a conserved adaptive transcriptional program rather than drug-specific effects.

To further investigate the potential common or specific biological functions of core resistance-associated DEGs under different drug treatments, this study conducted a systematic comparative analysis of the cellular resistance mechanisms induced by multiple drug categories. Overall, the enrichment results of different drug categories exhibited distinct commonalities in immune regulation, metabolic reprogramming, and extracellular microenvironment remodeling. This suggests that drug tolerance is not solely caused by changes in specific targets but rather arises from a coordinated adaptation of metabolic, signaling, and immune networks following exposure to different drugs, ultimately forming a conserved adaptive defense mechanism. KEGG pathways such as Pentose and glucuronate interconversions, Fat digestion and absorption, and complement and coagulation cascades were significantly enriched across multiple drug categories, indicating that these metabolism- and immunity-related pathways may mediate diverse resistance processes. Meanwhile, GO analysis revealed significant enrichment of terms such as vesicle lumen, endoplasmic reticulum lumen, outer membrane, mitochondrial outer membrane, collagen-containing extracellular matrix, blood microparticle, and extracellular matrix binding, all supporting the broad involvement of material transport, vesicle secretion, and extracellular matrix remodeling that facilitate the formation of resistance. This cross-drug coordination suggests that resistance may be governed by conserved cellular programs, rather than solely by the molecular targets of individual drugs. Collectively, these phenomena constitute the molecular basis of multidrug resistance.

Except for potential common regulatory patterns, drug-resistant cells induced by different categories of drugs also exhibited distinct molecular regulatory features. In Antibacterial Agents, pathways related to immune response, metabolic regulation, and signal transduction were significantly enriched. The upregulation of apoptosis pathways suggests that programmed cell death may contribute to the formation of antibacterial drug resistance. Previous studies have shown that some pathogens can evade immune clearance by modulating host cell apoptosis, thereby gaining a survival advantage. In Antineoplastic Agents, the significant enrichment of the Glycolysis/Gluconeogenesis pathway supports the “Warburg effect” in cancer cells, indicating that enhanced glycolysis allows cancer cells to maintain energy supply and mitigate metabolic stress induced by chemotherapeutic drugs [28]. This metabolic adaptation also reduces cellular sensitivity to oxidative stress, thereby decreasing drug-induced cytotoxicity and sustaining resistance. In Non-Steroidal Anti-Inflammatory Agents, enrichment of the drug metabolism–cytochrome P450 pathway highlights the central role of CYP450 metabolic enzymes in drug tolerance. CYP450 can accelerate drug metabolism, lower effective drug concentration, weaken drug efficacy, and contribute to resistance [29]. In Enzyme Inhibitors, the Apoptosis pathway was significantly enriched, consistent with previous studies. In some cell lines, overexpression of anti-apoptotic proteins (such as BCL-2) can block cell death induced by proteasome inhibitors (such as bortezomib or MG132), whereas combined treatment with TRAIL can restore sensitivity [30]. These observations indicate that resistance is closely associated with anti-apoptotic capability.

While our study demonstrates that machine learning-based analysis of core genes can capture key features of drug resistance and provides a framework for robust classification, all analyses were performed on a single dataset. Consequently, the generalizability of the findings remains to be confirmed. Future studies are warranted to validate these results in independent datasets and experimental systems, including in vitro and in vivo models, which will further strengthen the claims of broad applicability and biological relevance.

## 4. Materials and Methods

### 4.1. Data Acquisition and Preprocessing

Gene expression and drug sensitivity data were obtained from the DepMap database (https://depmap.org/portal/, accessed on 18 January 2026). Drug sensitivity, measured as log_2_ fold change (log_2_FC), where log_2_FC < 0 indicated that the cell line was sensitive to the compound, and log_2_FC ≥ 0 indicated resistance. Cell line identifiers were harmonized across datasets using a conversion table to enable integration of drug sensitivity profiles with gene expression data.

Drug classification was curated from PubChem (https://pubchem.ncbi.nlm.nih.gov/, accessed on 18 January 2026), yielding category annotations for 2100 compounds. Multi-label annotations were split into individual categories for frequency analysis, and only categories with ≥6 compounds were retained, resulting in 82 drug classes (1738 compounds). For subsequent analyses, only drugs with complete sensitivity data and sufficient numbers of both sensitive and resistant samples were included.

### 4.2. Identification of Differentially Expressed Genes

Differential gene expression analysis was performed on drug-treated cell line expression data using the DESeq2 package (version 1.46.0) in R. Genes with |log_2_FoldChange| ≥ 1 and adjusted *p*-value (*p*.adjust) < 0.05 were defined as significantly differentially expressed. For heatmap visualization, 500 samples and 500 genes were randomly selected; expression values were log_2_-transformed with a pseudocount of 1 and subsequently z-score normalized across genes. This random sampling strategy was adopted to reduce visual complexity while preserving the overall expression patterns and clustering structure of the dataset. Volcano plots and heatmaps were generated using ggplot2 (version 3.5.2) and pheatmap (version 1.0.12), respectively, providing an overview of gene expression profiles under different drug treatments. Hierarchical clustering was performed using Euclidean distance, and clusters were defined through dynamic tree cutting with the hybrid method (deepSplit = 2), enabling the identification of robust gene and sample groups.

### 4.3. Core Gene Selection

#### 4.3.1. Intersection Filtering of Differentially Expressed Genes

For each of the 82 drug categories, the intersection of DEGs across all compounds within the category was computed, applying an appropriate threshold to define the inclusion criteria for intersecting DEGs. UpSet plots depicting the number of intersecting genes across different drug categories and individual drugs were generated using the UpSetR package (version 1.4.0) in R.

#### 4.3.2. Core Gene Selection Using Machine Learning

In this study, three classical feature selection methods (Random Forest, Genetic Algorithm, and RFE) were employed to rank the intersected differentially expressed genes, thereby identifying core genes closely associated with drug resistance. The Random Forest-based feature selection was implemented using the RandomForestClassifier module from the Scikit-learn (version 1.3.0) library. The model was iterated 1000 times, with 500 trees constructed in each iteration, and the average feature importance values were calculated to generate the final ranking. The dataset was split into training and testing sets at a 7:3 ratio using train_test_split to obtain robust feature importance rankings.

The Genetic Algorithm used intersected differentially expressed genes as features and cell sensitivity classification labels as the target variable. Candidate solutions were encoded as binary vectors, and performance was evaluated on an independent test set using a Random Forest classifier from Scikit-learn (version 1.3.0). The evolutionary process involved tournament selection, crossover, and mutation operations, with computation accelerated by multiprocessing. Gene importance was determined based on their frequency during the evolutionary process.

RFE combined with the XGBoost classifier (XGBClassifier, xgboost version 2.1.4) was applied to perform feature selection on the gene expression data. Parallel computation was used to iteratively eliminate features until only the most representative features were retained.

To obtain a stable and objective gene importance ranking, the rankings produced by the three feature selection methods were integrated using a rank-based averaging strategy, in which each method contributed equally to the final comprehensive ranking. This integration approach reduces method-specific bias and enhances the robustness of the selected gene set. A circular heatmap of the top-ranked genes was generated using the R packages circlize (version 0.4.10) and ComplexHeatmap (version 2.22.0).

### 4.4. Construction of Resistance Classification Models

Differential expression data were standardized by Z-score normalization, and class imbalance was addressed using SMOTE oversampling (or optional undersampling). Model hyperparameters were optimized through grid search with five-fold cross-validation, without a separate test set, using ROC AUC as the evaluation criterion. For each fold, predicted probabilities and class labels were generated, feature importance was ranked based on the Random Forest-inherent Gini importance, and model performance was assessed using ROC and PR curves as well as confusion matrices, visualized with ggplot2 (version 3.5.2) and reshape2 (version 1.4.4).

### 4.5. Construction of PPI Networks

For further investigation of core DEGs, PPI networks were constructed for genes within each of the 46 drug categories, and network analysis methods were applied to identify the core genes. Within each category, drug-resistance-related genes were selected if they appeared in at least 60% of the drugs. The corresponding PPI data were obtained from the STRING database, and networks were visualized in Cytoscape (version 3.10.3), with node Degree values used for visualization. Topological analysis was then performed to calculate betweenness centrality, which was used to rank the genes. Genes with betweenness centrality above the median were defined as the core genes for the respective drug category.

### 4.6. GO/KEGG Enrichment Analysis

GO analysis and KEGG pathway enrichment analysis were conducted on the significantly differentially expressed genes of 82 drug categories using the R package clusterProfiler (version 4.14.6), and KEGG pathway–gene networks were constructed using igraph (version 2.1.4). A relatively relaxed threshold was used for GO and KEGG enrichment analyses to obtain a sufficient number of pathways for visualization. The results were then visualized with ggplot2 (version 3.5.2) and ggraph (version 2.2.1).

## 5. Conclusions

In this study, we systematically analyzed transcriptomic profiles of cell lines treated with thousands of drugs. The broad coverage and large-scale datasets ensured the reliability and generalizability of the results across different drug categories and mechanisms of action. Cross-drug comparisons further revealed potential common molecular bases underlying resistance mechanisms at a systematic level. Methodologically, by balancing stringency and inclusiveness in intersection thresholds and integrating multiple machine learning algorithms for core gene selection, we reinforced both the robustness and reliability of the findings. In addition, a stepwise multi-dimensional validation framework—including differential gene identification, core gene screening, classification modeling, PPI network construction, and GO/KEGG enrichment analysis—strengthened the interpretability of the results from both statistical and biological perspectives. Although these analyses still require further experimental validation, this study advances the comprehensive understanding of resistance mechanisms and provides a theoretical foundation for developing personalized therapeutic strategies and anti-resistance targets.

## Figures and Tables

**Table 1:** Core genes of PPI network.

Drug Categories	Core Genes
Antibacterial agents	*A2M*, *ALB*, *ELN*, *FGA*, *ADCYAP1*, *NTS*, *CHGA*, *SOX10*, *PRF1*, *PLA2G2A*, *MFAP4*
Antineoplastic agents	*A2M*, *ELN*, *FGA*, *ADCYAP1*, *NTS*, *ASCL1*, *CHGA*, *MFAP4*
Non-steroidal agents	*A2M*, *APOB*, *ALB*, *ELN*, *ITIH2*, *ADCYAP1*, *NTS*, *GRP*, *CHGA*, *GAPDHS*, *BGN*
Enzyme inhibitors	*A2M*, *APOB*, *ALB*, *ELN*, *ADCYAP1*, *NTS*, *CHGA*, *SOX10*, *PLA2G2A*, *MFAP4*

## Data Availability

The scripts implementing the three machine learning-based feature selection methods, together with the resistance classification models used in this work, have been made publicly accessible at https://github.com/YanwenWang23/TranDR-ML, accessed on 18 January 2026.

## Supplementary Materials

The following supporting information can be downloaded at: https://www.mdpi.com/article/10.3390/ijms27031245/s1.

## Abbreviations

The following abbreviations are used in this manuscript:

PPI	protein–protein interaction
RFE	Recursive Feature Elimination

## References

[1] Haider T., Pandey V., Banjare N., Gupta P.N., Soni V. (2020). Drug Resistance in Cancer: Mechanisms and Tackling Strategies. Pharmacol. Rep..

[2] Huemer M., Mairpady Shambat S., Brugger S.D., Zinkernagel A.S. (2020). Antibiotic Resistance and Persistence-Implications for Human Health and Treatment Perspectives. EMBO Rep..

[3] Housman G., Byler S., Heerboth S., Lapinska K., Longacre M., Snyder N., Sarkar S. (2014). Drug Resistance in Cancer: An Overview. Cancers.

[4] Antimicrobial Resistance Collaborators (2022). Global Burden of Bacterial Antimicrobial Resistance in 2019: A Systematic Analysis. Lancet.

[5] Walsh T.R., Gales A.C., Laxminarayan R., Dodd P.C. (2023). Antimicrobial Resistance: Addressing a Global Threat to Humanity. PLoS Med..

[6] Naghavi M., Vollset S.E., Ikuta K.S., Swetschinski L.R., Gray A.P., Wool E.E., Aguilar G.R., Mestrovic T., Smith G., Han C. (2024). Global Burden of Bacterial Antimicrobial Resistance 1990–2021: A Systematic Analysis with Forecasts to 2050. Lancet.

[7] Blair J.M.A., Webber M.A., Baylay A.J., Ogbolu D.O., Piddock L.J.V. (2015). Molecular Mechanisms of Antibiotic Resistance. Nat. Rev. Microbiol..

[8] Hu T., Li Z., Gao C.-Y., Cho C.H. (2016). Mechanisms of Drug Resistance in Colon Cancer and Its Therapeutic Strategies. World J. Gastroenterol..

[9] Efflux-Mediated Drug Resistance in Bacteria: An Update—PMC. https://pmc.ncbi.nlm.nih.gov/articles/PMC2847397/.

[10] Zhang S., Zhong X., Yuan H., Guo Y., Song D., Qi F., Zhu Z., Wang X., Guo Z. (2020). Interfering in Apoptosis and DNA Repair of Cancer Cells to Conquer Cisplatin Resistance by Platinum(IV) Prodrugs. Chem. Sci..

[11] Kadioglu O., Saeed M., Mahmoud N., Azawi S., Mrasek K., Liehr T., Efferth T. (2021). Identification of Potential Novel Drug Resistance Mechanisms by Genomic and Transcriptomic Profiling of Colon Cancer Cells with P53 Deletion. Arch. Toxicol..

[12] Wang N., Ma T., Yu B. (2023). Targeting Epigenetic Regulators to Overcome Drug Resistance in Cancers. Signal Transduct. Target. Ther..

[13] Antimicrobial Tolerance and Metabolic Adaptations in Microbial Biofilms—PubMed. https://pubmed.ncbi.nlm.nih.gov/31178124/.

[14] Metabolic Reprogramming and Therapeutic Resistance in Primary and Metastatic Breast Cancer|Molecular Cancer|Full Text. https://molecular-cancer.biomedcentral.com/articles/10.1186/s12943-024-02165-x.

[15] Zhu Y., Yan W., Tong L., Yang J., Ge S., Fan J., Jia R., Wen X. (2025). Metabolic Reprogramming: A Crucial Contributor to Anticancer Drug Resistance. MedComm.

[16] Chifiriuc M.C., Filip R., Constantin M., Pircalabioru G.G., Bleotu C., Burlibasa L., Ionica E., Corcionivoschi N., Mihaescu G. (2022). Common Themes in Antimicrobial and Anticancer Drug Resistance. Front. Microbiol..

[17] Boolchandani M., D’Souza A.W., Dantas G. (2019). Sequencing-Based Methods and Resources to Study Antimicrobial Resistance. Nat. Rev. Genet..

[18] Wang J., Dean D.C., Hornicek F.J., Shi H., Duan Z. (2019). RNA Sequencing (RNA-Seq) and Its Application in Ovarian Cancer. Gynecol. Oncol..

[19] Jiang J., Ma Y., Yang L., Ma S., Yu Z., Ren X., Kong X., Zhang X., Li D., Liu Z. (2025). CTR-DB 2.0: An Updated Cancer Clinical Transcriptome Resource, Expanding Primary Drug Resistance and Newly Adding Acquired Resistance Datasets and Enhancing the Discovery and Validation of Predictive Biomarkers. Nucleic Acids Res..

[20] DRUG-Seq for Miniaturized High-Throughput Transcriptome Profiling in Drug Discovery|Nature Communications. https://www.nature.com/articles/s41467-018-06500-x?utm_source=chatgpt.com.

[21] Park Y.H., Im S.-A., Park K., Wen J., Lee K.-H., Choi Y.-L., Lee W.-C., Min A., Bonato V., Park S. (2023). Longitudinal Multi-Omics Study of Palbociclib Resistance in HR-Positive/HER2-Negative Metastatic Breast Cancer. Genome Med..

[22] Huo Y., Shao S., Liu E., Li J., Tian Z., Wu X., Zhang S., Stover D., Wu H., Cheng L. (2022). Subpathway Analysis of Transcriptome Profiles Reveals New Molecular Mechanisms of Acquired Chemotherapy Resistance in Breast Cancer. Cancers.

[23] Li Y., Umbach D.M., Krahn J.M., Shats I., Li X., Li L. (2021). Predicting Tumor Response to Drugs Based on Gene-Expression Biomarkers of Sensitivity Learned from Cancer Cell Lines. BMC Genom..

[24] Sakagianni A., Koufopoulou C., Feretzakis G., Kalles D., Verykios V.S., Myrianthefs P., Fildisis G. (2023). Using Machine Learning to Predict Antimicrobial Resistance―A Literature Review. Antibiotics.

[25] Kim J.I., Maguire F., Tsang K.K., Gouliouris T., Peacock S.J., McAllister T.A., McArthur A.G., Beiko R.G. (2022). Machine Learning for Antimicrobial Resistance Prediction: Current Practice, Limitations, and Clinical Perspective. Clin. Microbiol. Rev..

[26] Chawla S., Rockstroh A., Lehman M., Ratther E., Jain A., Anand A., Gupta A., Bhattacharya N., Poonia S., Rai P. (2022). Gene Expression Based Inference of Cancer Drug Sensitivity. Nat. Commun..

[27] Firoozbakht F., Yousefi B., Schwikowski B. (2021). An Overview of Machine Learning Methods for Monotherapy Drug Response Prediction. Brief. Bioinform..

[28] Derouane F., Desgres M., Moroni C., Ambroise J., Berlière M., Van Bockstal M.R., Galant C., van Marcke C., Vara-Messler M., Hutten S.J. (2024). Metabolic Adaptation towards Glycolysis Supports Resistance to Neoadjuvant Chemotherapy in Early Triple Negative Breast Cancers. Breast Cancer Res..

[29] Thistlethwaite S., Jeffreys L.N., Girvan H.M., McLean K.J., Munro A.W. (2021). A Promiscuous Bacterial P450: The Unparalleled Diversity of BM3 in Pharmaceutical Metabolism. Int. J. Mol. Sci..

[30] Nencioni A., Wille L., Dal Bello G., Boy D., Cirmena G., Wesselborg S., Belka C., Brossart P., Patrone F., Ballestrero A. (2005). Cooperative Cytotoxicity of Proteasome Inhibitors and Tumor Necrosis Factor-Related Apoptosis-Inducing Ligand in Chemoresistant Bcl-2-Overexpressing Cells. Clin. Cancer Res..

