# Supplementary material for: Machine Learning-Based Analysis of Large-Scale Transcriptomic Data Identifies Core Genes Associated with Multi-Drug Resistance
Source: Int J Mol Sci. 2026 Jan 27;27(3):1245. doi: 10.3390/ijms27031245 (PMC12898147; doi:10.3390/ijms27031245)
Supplement: Supplementary file 1 [file ijms-27-01245-s001.zip › Supplementary Figure.pdf]

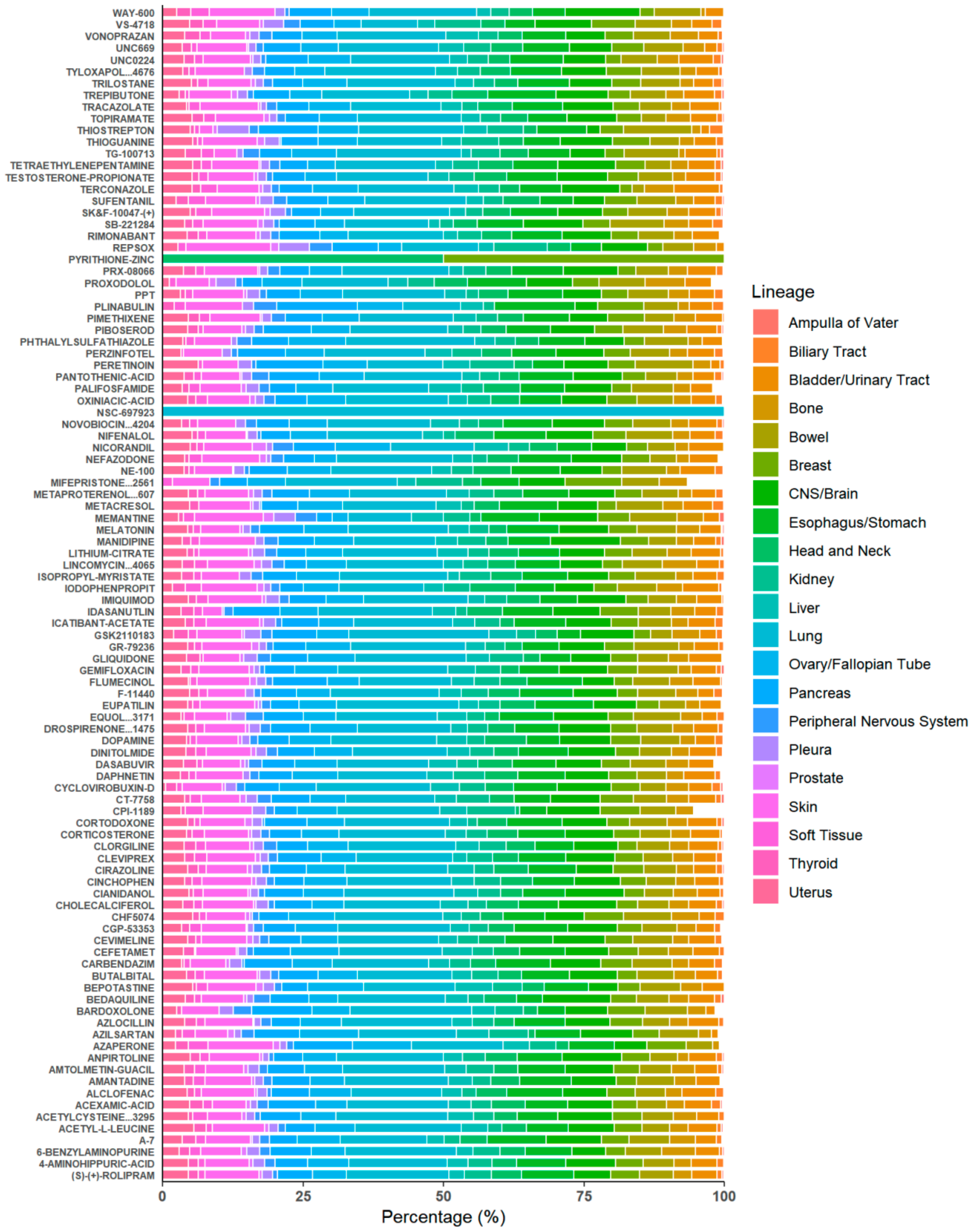

Figure S1. Lineage composition of resistant cells for each drug. Horizontal stacked bar plot showing lineage composition of resistant cells for each drug. Colors indicate tissue lineages; the y-axis lists drugs, and the x-axis shows the percentage of each lineage. Minor gaps are due to rounding.

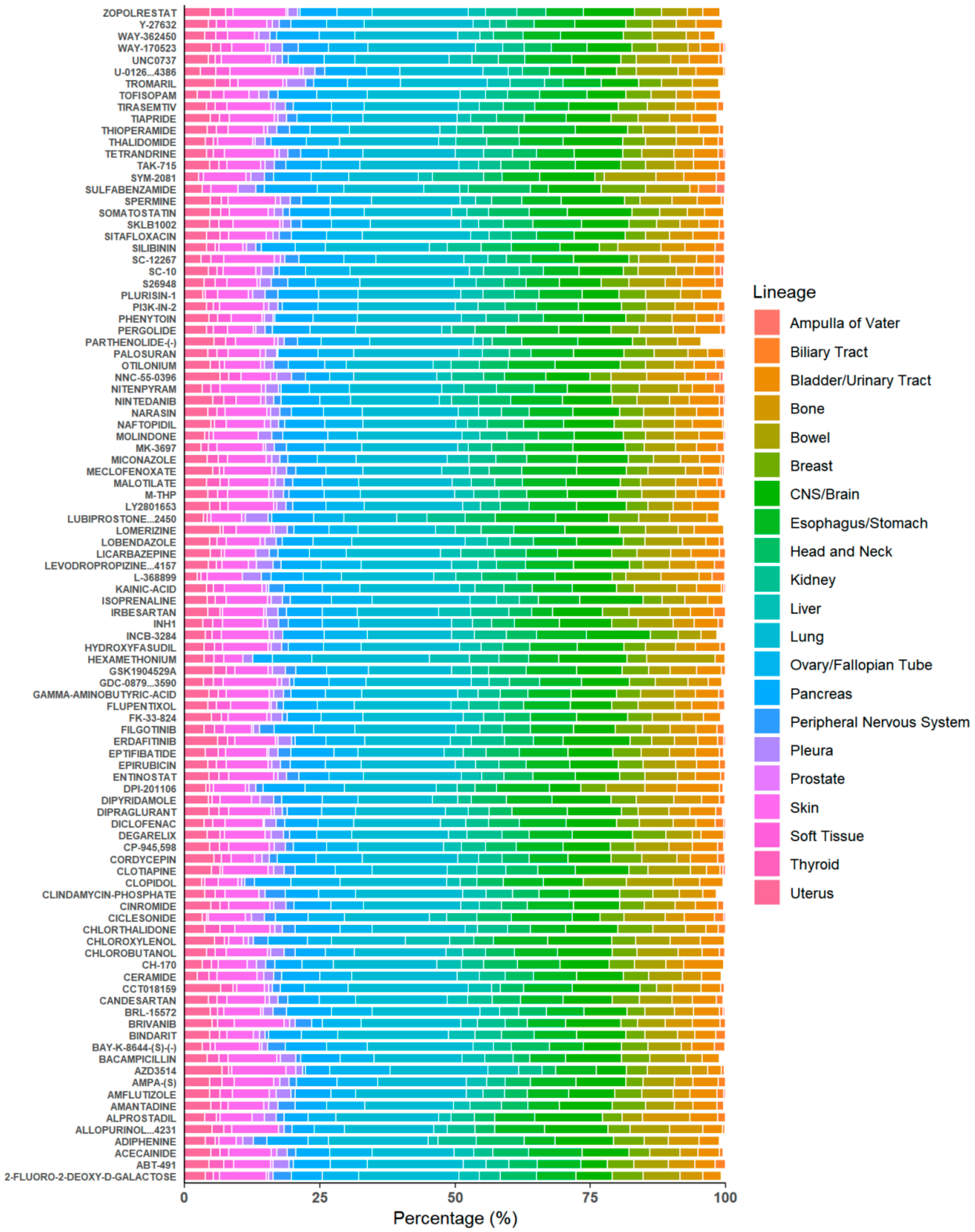

Figure S2. Lineage composition of sensitive cells for each drug. Horizontal stacked bar plot showing lineage composition of sensitive cells for each drug. Colors indicate tissue lineages; the y-axis lists drugs, and the x-axis shows the percentage of each lineage. Minor gaps are due to rounding.

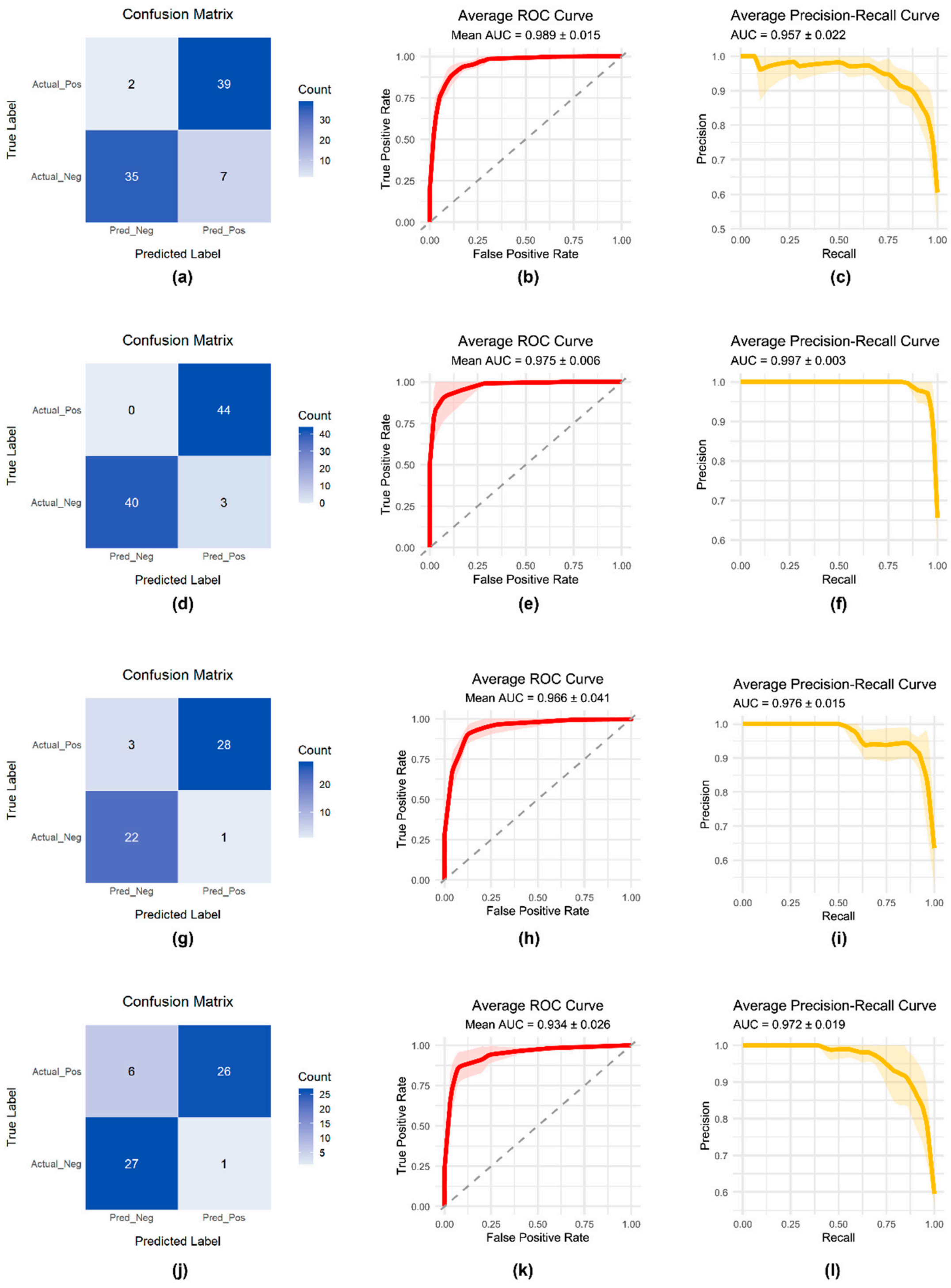

Figure S3. Resistance prediction performance of the model across multiple drugs. Oxaliplatin (Antineoplastic Agents, a–c), Nafcillin (Anti-Bacterial Agents, d–f), Methotrexate (Enzyme Inhibitors, g–i), and Phenylbutazone (Non-Steroidal Anti-Inflammatory Agents, j–l) are shown. For each drug: a/d/g/j, confusion matrix of classification results; b/e/h/k, Receiver Operating Characteristic (ROC) curve; c/f/i/l, Precision–Recall (PR) curve. This figure extends the analysis presented in the main text (Salinomycin) to multiple pharmacological categories, providing a broader view of resistance prediction across different drugs.
